# Key inflammatory mechanisms underlying heart failure

**DOI:** 10.1007/s00059-019-4785-8

**Published:** 2019-02-04

**Authors:** C. Riehle, J. Bauersachs

**Affiliations:** Department of Cardiology and Angiology, Hannover Medical School, Carl-Neuberg-Str. 1, 30625 Hannover, Germany

**Keywords:** Cardiac failure, Inflammation, Myocardial infarction, Immune system, Cytokines, Herzinsuffizienz, Entzündung, Myokardinfarkt, Immunsystem, Zytokine

## Abstract

Inflammation plays a central role in the development of heart failure, especially in heart failure with preserved ejection fraction (HFpEF). Furthermore, the inflammatory response enables the induction of regenerative processes following acute myocardial injury. Recent studies in humans and animals have greatly advanced our understanding of the underlying mechanisms behind these adaptations. Importantly, inflammation can have both beneficial and detrimental effects, dependent on its extent, localization, and duration. Therefore, modulation of cardiac inflammation has been suggested as an attractive target for the treatment of heart failure, which has been investigated in numerous clinical trials. This review discusses key inflammatory mechanisms contributing to the pathogenesis of heart failure and their potential impact as therapeutic targets.

## Zusammenfassung

Entzündungsprozesse spielen eine zentrale Rolle bei der Entwicklung der Herzinsuffizienz, insbesondere bei Herzinsuffizienz mit erhaltener Ejektionsfraktion (HFpEF). Darüber hinaus sind Entzündungsprozesse allerdings auch für die Reparationsvorgänge nach akutem Myokardinfarkt erforderlich. Sowohl aktuelle Studien an Tiermodellen als auch Untersuchungen an Menschen führten zu einem besseren Verständnis der zugrunde liegenden Mechanismen. Abhängig von Lokalisation, Ausmaß und der Dauer können Entzündungsprozesse sowohl vorteilhaft als auch nachteilig sein. Deshalb bietet sich deren Beeinflussung als ein möglicher Angriffspunkt zur Behandlung der Herzinsuffizienz sowie pathologischer Umbauvorgänge an. Dies ist Gegenstand zahlreicher klinischer Studien. In der vorliegenden Übersichtsarbeit wird die Rolle wesentlicher Entzündungsprozesse in der Pathogenese der Herzinsuffizienz erörtert und deren potenzielle Bedeutung als Therapieoption diskutiert.

Heart failure (HF) is a clinical syndrome based primarily on systolic or diastolic left-ventricular (LV) contractile dysfunction. The prognosis of chronic HF is poor, with about 50% of patients dying within 5 years after the initial diagnosis. There are different categories of HF, which are based on measurements of LV ejection fraction (LVEF). About half of HF patients are afflicted with HF with reduced ejection fraction (HFrEF) with an LVEF of <40%. In contrast, HF with preserved ejection fraction (HFpEF) is observed in roughly the other half of patients (LVEF ≥50%). Patients with an LVEF in the range of 40–49% represent a “gray area” that is defined as HF with mid-range ejection fraction (HFmrEF; [1]). The prevalence of HF in industrialized nations is increasing to more than 10% among people greater 70 years of age [2]. Statistically, about one in three individuals at 55 years of age will develop HF during their remaining lifespan [3]. The increase in HF can be explained by the rising prevalence of renal failure, arterial hypertension, chronic obstructive pulmonary disease (COPD), diabetes mellitus, and metabolic syndrome. These comorbidities are characterized by chronic inflammation and are of particular importance for patients with HFpEF [2]. Furthermore, the treatment of ischemic heart disease has significantly improved over the past few decades, which has increased the number of surviving HF patients.

In addition to playing a critical role in the development and progression of HFpEF and HFrEF [4, 5], the inflammatory response is also important for adverse remodeling processes following myocardial infarction (MI). The development of HF can also be directly immune-modulated, for example, following autoimmune or infectious triggers, i. e., viral infection. Following acute myocardial injury, the inflammatory response is required to induce the regenerative response, but sustained and chronic inflammation is detrimental. Based on the dichotomous role of inflammation in cardiac tissue, the modulation of inflammatory processes has been identified as a therapeutic approach. The pathomechanisms underpinning inflammation modulation for therapeutic benefit have been investigated in numerous studies and will be summarized in this review.

## HFpEF, endothelial dysfunction, and inflammation

**Fig. 1:**
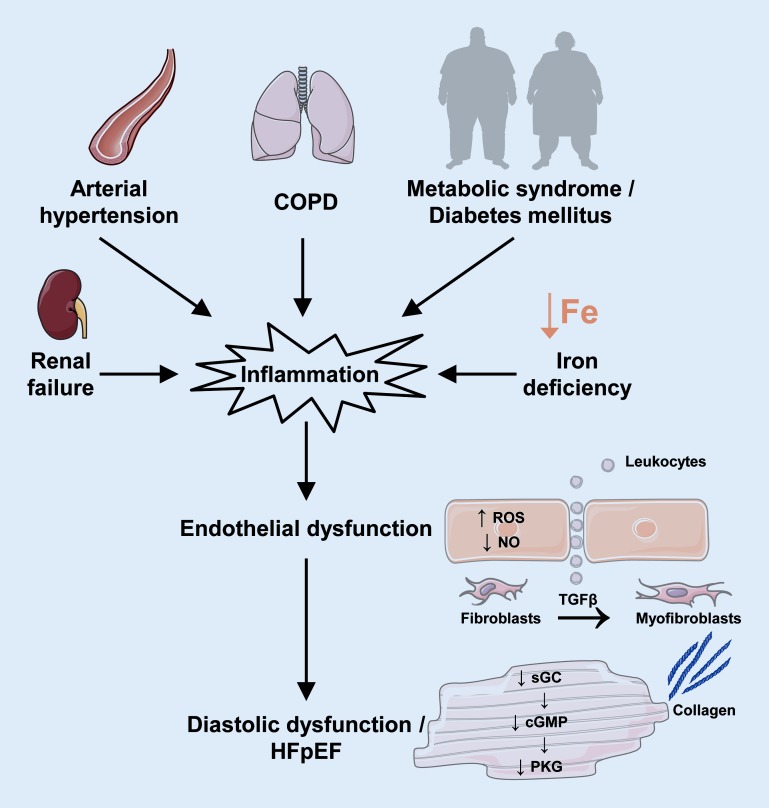
Schematic depicting the impact of endothelial dysfunction and inflammation on the development of fibrosis and heart failure with preserved ejectionfraction (*HFpEF*). Comorbidities, such as renal failure, arterial hypertension, chronic obstructive pulmonary disease (*COPD*), metabolic syndrome, diabetes mellitus, and iron deficiency, induce systemic inflammation. Increased mitochondrial reactive oxygen species (*ROS*) production, increased peroxynitrite (*ONOO*^−^) levels, and decreased nitric oxide (*NO*) levels in endothelial cells attenuate cardiomyocyte soluble guanylate cyclase (*sGC*)/guanosine monophosphate (*cGMP*)/protein kinase G (*PKG*) signaling, which induces adverse left-ventricular remodeling and diastolic dysfunction. Inflammation also promotes fibrosis by differentiation of fibroblasts into myofibroblasts following transforming growth factor beta (*TGFβ*) secretion by monocytes

One hallmark of HFpEF is impaired LV relaxation as a consequence of altered composition of the extracellular matrix and decreased cyclic guanosine monophosphate (cGMP)/protein kinase G (PKG) signaling. From a mechanistic perspective, comorbidities promote systemic inflammation, which increases reactive oxygen species (ROS) production in cardiac endothelial cells and peroxynitrite (ONOO^−^) levels. The subsequent decrease in nitric oxide (NO) in endothelial cells impairs soluble guanylate cyclase (sGC) levels and PKG activity in adjacent cardiomyocytes. This promotes adverse LV remodeling and hypophosphorylation of titin, which impairs LV relaxation. Furthermore, monocytes infiltrate cardiac tissue under conditions of chronic inflammation and differentiate into macrophages, which augment myocardial inflammation. This also promotes fibrosis by differentiation of fibroblasts into myofibroblasts following transforming growth factor beta (TGF β) secretion by monocytes ([6]; Fig. 1).

Several studies provide mechanistic insight into the cardioprotective effects of NO/sGC/cGMP/PKG signaling. For example, pharmacological stimulation of sGC attenuates LV remodeling after MI in mice, decreases extracellular matrix protein production in human cardiac fibroblasts following TGFβ stimulation in vivo [7], and attenuates vascular dysfunction in diabetic rats [8]. Similarly, the endothelial NO synthase (eNOS) transcription enhancer AVE9488 improves cardiac remodeling after MI [9] and platelet NO availability and hyperactivity in HF [10]. Senescence-accelerated-prone mice (SAMP) develop manifest HFpEF when subjected to a high-salt, high-fat diet, which is characterized by endothelial cell dysfunction and fibrosis. These studies highlight the contribution of endothelial cell dysfunction on the age-dependent increase in HFpEF [11]. Furthermore, increased insulin-like growth factor-1 (IGF-1) activity following growth hormone stimulation attenuates age-dependent endothelial progenitor cell dysfunction [12]. Myeloperoxidase (MPO) is a bactericidal enzyme that is released from activated polymorphonuclear neutrophils and can directly modulate the vascular inflammatory response by regulating NO bioavailability [13]. Importantly, MPO also promotes HF following ischemic injury [14], atrial structural remodeling, and increases the risk of atrial fibrillation [15].

Mice with cardiomyocyte-specific deletion of iron-regulatory proteins (Irp) 1 and 2 exhibit mitochondrial dysfunction and accelerated HF after MI [16], which underscores the importance of iron availability in cardiomyocytes. This is further emphasized by impaired mitochondrial capacity and contractility in human embryonic stem cell-derived cardiomyocytes following incubation with the iron chelator deferoxamine. Mitochondrial capacity and contractility were restored following enhanced intracellular iron levels, suggesting that iron levels directly mediate these effects [17]. It has been shown that LV samples from failing human hearts exhibit decreased iron content, which may impair mitochondrial capacity and ROS scavenging in these samples [18]. ROS can mediate both beneficial and deleterious effects that are based on the subcellular localization and duration of exposure to ROS, as recently reviewed [19].

The importance of inflammation in the development of HFpEF has been demonstrated in a swine model following induction of the three most common inflammation-associated comorbidities in HFpEF patients: arterial hypertension, diabetes mellitus, and hypercholesterolemia [20]. Diabetes mellitus also increases the risk of diastolic dysfunction and HF independent of coexisting coronary artery disease and hypertension. This resulted in the term “diabetic cardiomyopathy.” Various mechanisms increase the risk of HF in diabetic patients [21, 22], including increased inflammation. The underlying mechanisms of inflammatory-dependent HF in diabetic patients include increased expression levels of interleukins (IL-1β, IL-6), intercellular adhesion molecule-1 (ICAM-1), and vascular cell adhesion molecule-1 (VCAM-1), and decreased activity of the collagen degrading matrix metalloproteinase (MMP).

Finally, autophagy is a highly conserved cellular process that plays important roles in the maintenance of cellular homeostasis and quality control of organelles. Depending on the extent and duration of autophagy, this cellular process can be both beneficial and detrimental [23, 24]. Perturbed cardiac autophagy has been described for several risk factors of HFpEF development, including hypertension, diabetes, and aging [25].

## Inflammation following ischemic injury

**Fig. 2:**
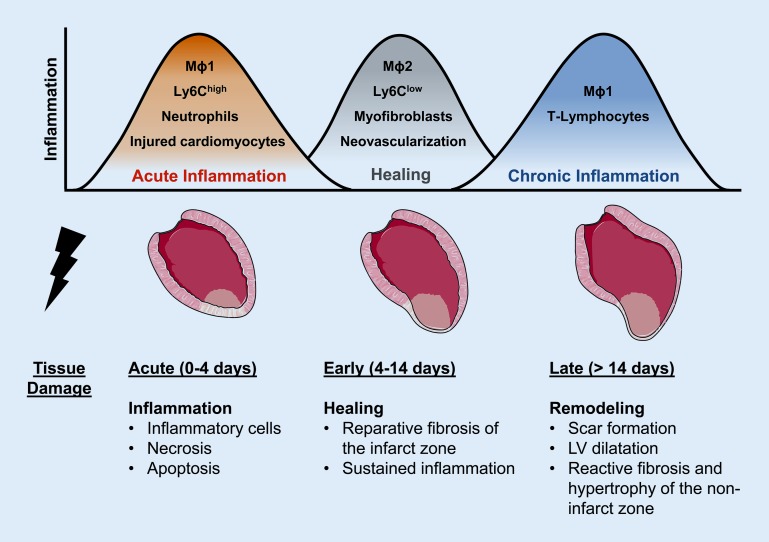
Time course of inflammation and healing after myocardial infarction in mice. The acute inflammatory response is characterized by infiltration with M1 macrophages (*Mɸ1*), Ly6C^high^ monocytes, and neutrophils. The main characteristics of the healing phase are infiltration with M2 macrophages (*Mɸ2*), Ly6C^low^ monocytes and myofibroblasts, which contribute to wound repair, neovascularization, limitation of tissue damage, and reparative fibrosis of the infarct zone. Chronic inflammation might result from persistent inflammation following the healing phase or a second boost of inflammation. Note that the categorization of macrophages into “M1” and “M2” subtypes is an oversimplification and that the different phases require a greater time span in larger animals and humans. *LV* left-ventricular

Macrophages and monocytes are essential for the inflammatory response and ventricular remodeling following ischemic injury [26, 27]. The cellular response to myocardial ischemia can be categorized into different phases: the acute inflammatory phase, the healing phase, and a phase of chronic inflammation. A schematic summary of these events after MI in mice is provided in Fig. 2. The different phases are well characterized in murine models and require a greater time span in larger animals and humans. The cellular and inflammatory adaptations are mediated by neutrophils and monocytes, which are generated in the bone marrow and the spleen and then translocate to the injured myocardium. Three monocyte subsets have been described in humans; these are based on the expression pattern of the surface protein expression markers CD14 and CD16. On the basis of these expression patterns, monocytes can be classified as classic (CD14^++^CD16^−^; murine homolog: Ly6C^high^), intermediate (CD14^++^CD16^+^), and nonclassic (CD14^+^CD16^++^; murine homolog: Ly6C^low^) monocytes.

Ly6C^high^ monocytes express high levels of Ly6C, CCR2, and CD62L and play a predominant role in the initial inflammatory phase after MI. Recruitment of Ly6C^high^ monocytes is mediated by high expression levels of the cytokine CCL2. Ly6C^high^ monocytes produce high levels of pro-inflammatory cytokines, such as IL-1β and tumor necrosis factor alpha (TNFα), which resulted in the term “inflammatory” monocytes for these cells. Ly6C^high^ monocytes recruit inflammatory macrophages, which secrete proteolytic enzymes to digest and prepare the damaged tissue for regeneration. Inflammatory macrophages are commonly termed “M1” macrophages. Decreasing the recruitment of neutrophils to the injured myocardium is essential in order to limit tissue injury and to initiate the healing process. Following ingestion of damaged and apoptotic cells, macrophages decrease their production of IL-1β and TNFα, and increase the secretion of anti-inflammatory and pro-fibrotic cytokines, i. e., IL-10 and TGFβ [28]. Following transformation and change of their activation profile, less inflammatory macrophages are termed “M2” or “reparative” macrophages. Neutrophils play a critical role in this polarization of macrophages toward a reparative M2 phenotype [29]. Although commonly used, the categorization of macrophages into “M1” and “M2” subtypes is an oversimplification to describe their heterogeneity, which is originally based on in vitro studies and is problematic for describing adaptations in vivo.

The second phase, called the “healing phase,” is dominated by Ly6C^low^ monocytes. These cells are recruited via CX_3_CR_1_ (fractalkine receptor)-mediated signaling to the infarcted region and are present at much lower numbers compared with Ly6C^high^ monocytes [30]. Ly6C^high^ can differentiate into Ly6C^low^ monocytes. However, the exact relationship between M1 and M2 macrophages and Ly6C^high^ and Ly6C^low^ monocytes requires further investigation [31]. A seminal study performed by the Molkentin group greatly improved our understanding of how myocardial scar tissue forms after ischemic injury [32]. Using transgenic lineage-tracing mouse lines as reporter constructs, the authors show that both cardiomyocytes and fibroblasts die in the infarcted area. Subsequently, fibroblasts from the border zone region are activated and proliferate greatly, which results in an approximately 3.5-fold increase in the total number of fibroblasts in the infarcted area relative to uninjured conditions 3 days after MI. This elevation in count was observed for over 4 weeks. Between days 3 and 7, fibroblasts differentiate into myofibroblasts, as indicated by smooth muscle α-actin expression. Subsequently, the proliferation of myofibroblasts and smooth muscle α-actin expression decrease by days 7–10, while the scar tissue matures.

Following the healing phase, inflammation often persists or reoccurs during the development of HF. Hallmarks of chronic inflammation are the increased abundance of tissue T‑lymphocytes and pro-inflammatory M1 macrophages [33, 34]. Risk factors for the development of HF include an initial inflammatory response [35] and large MI [36]. A recent study showed that the lymphatic system is required for clearing immune cells and limiting the immune response in cardiac tissue following ischemic injury. Genetic deletion of lymphatic vessel endothelial hyaluronan receptor 1 (LYVE-1) in mice decreased the clearance of leukocytes to mediastinal lymph nodes following MI [37]. This resulted in increased pathological remodeling and decreased cardiac function. These intriguing data emphasize the adverse effect of persistent inflammation following MI.

The risk of acute atherothrombotic events is increased following MI, both at culprit and nonculprit arteries [38]. Several changes in remote vessels following MI may contribute to accelerated plaque formation and inflammation: increased platelet-endothelial adhesion from endothelial-associated von Willebrand factor (VWF) multimers and increased expression of endothelial inflammatory adhesion molecules [39]. Even though the underlying molecular mechanisms require further investigation, these observations suggest increased inflammation is associated with an increased risk of future ischemic events following MI. Similarly, MI increases the risk of atherosclerosis [40]. A recently identified target for cardiac regeneration after MI is glucocorticoid receptor (GR) expression in macrophages. GR regulates factors that control inflammation and neovascularization, which are required for the preservation of contractile function and scar tissue formation [41]. Monocytes and macrophages are also important for healing and for the prevention of ventricular thrombus formation after MI [42].

Multimodality noninvasive imaging has been used to assess the inflammatory response in patients following MI and identified the spleen and bone marrow as sources of inflammatory cells [43]. Using positron emission tomography (PET), it has also been shown that acute myocardial inflammation predicts subsequent functional outcome and neuroinflammation after MI [44]. The mechanisms contributing to chronic inflammation are incompletely understood and include persistent inflammation following the healing phase and resurgence in inflammation [45]. Stem cell transplantation following MI has been suggested as a promising therapeutic approach to limit tissue damage and preserve contractile function. While most of the transplanted cells die from apoptosis and contribute little to neovascularization, these cells may also mediate the immune response resulting in reduced scar tissue formation and improved cardiac outcome (“dying stem cell hypothesis”; [46]).

## Mineralocorticoid receptor-mediated signaling

The mineralocorticoid aldosterone mediates inflammatory pathways and is critical for adverse cardiac remodeling. Aldosterone is produced in the glomerular zone of the adrenal cortex and increases sodium reabsorption, potassium secretion, and blood pressure. This is facilitated following its binding to the mineralocorticoid receptor (MR), which is a member of the nuclear receptor transcription factor family. Following translocation to the nucleus and homodimerization, MRs promote the expression of target genes. MRs are also expressed in various cell types of the heart and the vasculature, including cardiomyocytes, fibroblasts, coronary endothelial cells, vascular smooth muscle cells, and inflammatory cells [47]. Myocardial MR expression is increased in patients with HF [48]. MR antagonists reduced mortality and morbidity rates in patients with HF in large clinical studies (RALES, EPHESUS and EMPHASIS-HF trials [49–51]) and are commonly used as a standard treatment for patients with HF. The EPHESUS trial also showed that early initiation of MR blockage after MI and concomitant HF is beneficial relative to later initiation of the treatment [52]. A prespecified meta-analysis of the ST-segment elevation myocardial infarction (STEMI) subgroup of the ALBATROSS and the REMINDER trials shows that early initiation of MR antagonist treatment in patients with STEMI reduces mortality and the composite of death or resuscitated sudden cardiac death [53]. These data highlight the benefits of MR antagonist treatment following MI.

Aldosterone and MR signaling promote inflammation, myocardial hypertrophy, adverse LV remodeling, and ischemic injury. These effects are, at least in part, independent of systemic blood pressure and transduced by increased pro-inflammatory and pro-fibrotic signaling, i. e., TNFα, TGFβ, connective tissue growth factor (CTGF), and increased oxidative stress induced by NADPH oxidases [54–56]. Several studies using mouse models have advanced our understanding of cardiac MR-mediated signaling. Mice with genetic deletion of the MR in myeloid cells are protected against cardiac hypertrophy, fibrosis, and vascular damage induced by L‑NAME/angiotensin II treatment. Furthermore, these mice exhibit an alternatively activated M2 macrophage phenotype. This indicates that MR expression in myeloid cells is required for efficient classic macrophage activation by pro-inflammatory cytokines [57]. Genetic deletion of the MR in cardiomyocytes, but not in fibroblasts, attenuates contractile dysfunction and HF following pressure overload induced by transverse aortic constriction. However, MR deletion in cardiomyocytes or fibroblasts has no impact on cardiac fibrosis and hypertrophy relative to wild type controls following pressure overload [58]. This suggests a potential predominant role of MR expression in myeloid cells in this context.

A series of studies performed by our laboratory has identified several MR-mediated cardioprotective mechanisms following MI. Treatment with the MR antagonist eplerenone attenuates adverse LV remodeling and contractile dysfunction in rats. The underlying mechanisms include accelerated macrophage infiltration, a transient increase in protective cytokines, and alternative M2 macrophage activation [59]. In this context, treatment with eplerenone is superior relative to spironolactone by increasing the abundance of healing Ly6C^low^ monocytes and neovessel formation [60]. Additional mechanistic insight is provided by a transgenic mouse model with cardiomyocyte-specific MR deletion, which exhibits increased healing and attenuated contractile dysfunction [61]. In this model, MR deletion reduces infarct expansion and myocyte apoptosis, while infarct neovessel formation is increased in the early phase after ischemic damage. Furthermore, oxidative stress in the surviving LV myocardium is attenuated. This inflammatory cellular response is accelerated with a transient infiltration of neutrophils, which improves neovascularization and attenuates pathological remodeling. We also observed decreased expression of the MR target gene serum/glucocorticoid-regulated kinase 1 (SGK1) in MR-deficient cardiomyocytes, which mediates cardiomyocyte hypertrophy by increasing CTGF expression [62]. Notably, myeloid cell-specific MR deficiency also attenuates LV dysfunction and LV remodeling following MI in mice by decreasing inflammation and oxidative stress [63]. Together, our studies identify a critical role of MR-transduced signaling to mediate tissue damage in ischemic heart injury. The mechanisms discovered include activation of inflammatory pathways in various cell types. Our studies also strongly support the importance of MR-antagonist treatment of patients with ischemic heart disease.

## Anti-inflammatory and immune-modulatory treatment of patients with HF

Several clinical trials have tested the impact of anti-inflammatory and immune-modulatory therapies in patients with myocarditis, inflammatory cardiomyopathy, and HF [64, 65]. Despite this promising therapeutic approach, these studies have provided ambiguous results (Table 1). Based on its potential contribution to the progression of HF, the pro-inflammatory cytokine TNFα was identified as a promising pharmaceutical target. The randomized placebo-controlled ATTACH [66] and RENEWAL [67] trials tested the impact of the chimeric TNFα-antibody infliximab and the TNFα-inhibitor etanercept, respectively. The data obtained show no advantage of these treatments in patients with HF. Moreover, the ATTACH study reports adverse effects of infliximab at higher doses [66]. Potential mechanisms for these observations include binding of infliximab to TNFα-expressing cardiomyocytes, which might induce complement activation and cardiomyocyte apoptosis. Furthermore, administration of relatively high doses of infliximab might suppress TNFα below physiological concentrations, which are cardioprotective in the context of acute ischemic injury [68]. Gullestad and colleagues [69] recently described beneficial effects of the sedative and antinausea drug thalidomide. Despite the previously reported anti-inflammatory effects of thalidomide, the mechanisms are not completely understood and may include matrix stabilization based on decreased MMP2 expression.

**Table 1:** Summary of major clinical trials targeting inflammatory pathways and immune-modulatory therapies in heart failure

Study	Treatment	Target	Duration (months)	Clinical setting	NYHA class	*n*	Primary outcome
ATTACH [66]	Infliximab	TNFα	7	DCM, ICM	III, IV	150	↑ Death and hospitalization for HF at high doses
RENEWAL (RECOVER and RENAISSANCE) [67]	Etanercept	TNFα	5.7/12.9	DCM, ICM	II–IV	2048	↔ Death and hospitalization rate for HF
Gullestad et al. [69]	Thalidomide	Multiple	3	DCM, ICM	II, III	56	↑ LVEF
Parrillo et al. [81]	Prednisone	Multiple	3	DCM	–	102	↑ LVEF
Skudicky et al. [70]	Pentoxifylline	Multiple	6	DCM	II, III	39	↑ LVEF and symptoms
Sliwa et al. [71]	Pentoxifylline	Multiple	6	DCM	II, III	28	↑ LVEF and symptoms
Sliwa et al. [72]	Pentoxifylline	Multiple	1	DCM	IV	18	↑ LVEF and ↓ TNFα
Sliwa et al. [73]	Pentoxifylline	Multiple	6	ICM	II, III	38	↑ LVEF and ↓ plasma inflammatory markers
Bahrmann et al. [74]	Pentoxifylline	Multiple	6	DCM, ICM	II, III	47	↔ LVEF
CORONA [75]	Rosuvastatin	Multiple	32.8	ICM	II–IV	5011	↔ Cardiovascular death, nonfatal MI, and nonfatal stroke
GISSI-HF [76]	Rosuvastatin	Multiple	46.9	DCM, ICM	II–IV	4574	↔ Death and cardiovascular hospitalization
Krum et al. [77]	Rosuvastatin	Multiple	6	DCM, ICM	II–IV	87	↔ LVEF
ACCLAIM [78]	Device-based immunomodulation	Nonspecific	10.2	DCM, ICM	II–IV	2426	↔ Death and cardiovascular hospitalization
Gullestad et al. [79]	Intravenous immunoglobulin	Multiple	6	DCM, ICM	II, III	40	↑ LVEF
IMAC [80]	Intravenous immunoglobulin	Multiple	12	DCM	I–IV	62	↔ LVEF
METIS [82]	Methotrexate	Multiple	3	ICM	II–IV	50	↔ 6‑Minute walk test

*ACCLAIM* Advanced Chronic Heart Failure Clinical Assessment of Immunomodulation, *ATTACH* Anti-TNF Therapy Against Congestive Heart Failure, *CORONA* Controlled Rosuvastatin Multinational Trial in Heart Failure, *DCM* dilated cardiomyopathy, *GISSI-HF* Gruppo Italiano per lo Studio della Sopravvivenza nell’Insufficienza cardiaca-Heart Failure, *HF* heart failure, *ICM* ischemic cardiomyopathy, *IMAC* Intervention in Myocarditis and Acute Cardiomyopathy, *LVEF* left-ventricular ejection fraction, *METIS* Methotrexate Therapy on the Physical Capacity of Patients with Ischemic Heart Failure, *MI* myocardial infarction, *RECOVER* Etanercept Cytokine Antagonism in Ventricular Dysfunction, *RENAISSANCE* Randomized Etanercept North American Strategy to Study Antagonism of Cytokines, *RENEWAL* Randomized Etanercept Worldwide Evaluation

Pentoxifylline is an anti-inflammatory agent that inhibits the production of TNFα and IL-6. Treatment with pentoxifylline increased LV contractile function and attenuates HF symptoms in some studies [70–73], while another report showed no difference [74]. Pentoxifylline is also a nonselective phosphodiesterase inhibitor. Therefore, pentoxifylline might mediate its cardioprotective effects by inhibiting phosphodiesterases and being, at least in part, independent of reducing inflammation. In addition to attenuating the formation of low-density lipoprotein by inhibiting the enzyme HMG-CoA reductase, statins are anti-inflammatory and improve endothelial function. However, treatment with statins is not beneficial in the context of HF unless administered in the presence of other comorbidities, such as dyslipidemia or coronary artery disease [75–77].

“Immunomodulation” therapy may provide a beneficial immune response to decrease pro-inflammatory and increase anti-inflammatory pathways. Patients with HF were subjected to “immunomodulation” by exposure of autologous blood ex vivo to controlled amounts of oxidative stress before administration by intragluteal injection. The ACCLAIM trial showed no impact of “immunomodulation” therapy on mortality and cardiovascular hospitalization [78]. Additional studies are required to understand the exact mechanisms of “immunomodulation” therapy that may contribute to potential positive effects of this treatment. In another study, HF patients were subjected to intravenous immunoglobulin (IVIg) infusions to modulate the immune response, which increased contractile function in patients with ischemic cardiomyopathy (ICM) and idiopathic dilated cardiomyopathy (DCM) [79]. By contrast, the IMAC trial [80] showed a similar increase in contractile function in patients with DCM or myocarditis who were treated with IVIg or placebo. It is important to note that the IMAC trial does not provide any data on histological sections from myocardial biopsies, inflammation, and viral persistence for a later time point. Thus, it is challenging to discern the potential benefits of IVIg therapy relative to standard HF therapy, which was administered to HF patients independent of IVIg or placebo. This is of particular importance for patients with myocarditis, who might benefit from the antiviral and immune-modulatory effects of IVIg therapy the most.

Additional therapeutic approaches to modulate the immune response in patients with HF include treatment with prednisone and methotrexate as well as by reduction in the abundance of auto-antibodies by immunoadsorption. In summary, the results of most studies targeting anti-inflammatory and immune-modulatory therapy are ambiguous. Table 1 summarizes major published clinical trials with anti-inflammatory and immune-modulatory treatment in patients with HF. Future research is warranted to identify additional targets for the modulation of inflammation in HF.

## Conclusion

Inflammation plays a central role in the development of the different etiologies of HF, especially in HFpEF. Importantly, the inflammatory response following ischemic damage is also required to induce the regenerative response and is transduced by MR-mediated signaling. Decreased MR signaling is beneficial following ischemic damage because of the attenuation of pathological remodeling and MR antagonists are a well-established standard treatment for HF. A variety of key inflammatory markers have been identified that have been subsequently tested as potential targets for the treatment of HF. Even though clinical trials have provided inconclusive results, modulation of inflammation remains a promising target for the treatment of HF. Additional studies are required to further delineate the mechanisms and to identify novel target molecules, which is the subject of ongoing research in this field.
